# A novel physical colonoscopy simulator based on analysis of data from computed tomography colonography

**DOI:** 10.1007/s00595-017-1517-7

**Published:** 2017-05-03

**Authors:** Katsuhisa Noda, Takatoshi Kitada, Yasumoto Suzuki, Hugh Shunsuke Colvin, Taishi Hata, Tsunekazu Mizushima

**Affiliations:** 1 Noda Genki Clinic, Kawasaki Building 1F 102, 2-6-30 Minami-Sakurazuka, Toyonaka, Osaka 561-0882 Japan; 2 Kitada Clinic., Geo Kawanishi Residence Mark 1F, 1-2-6 Ohbe, Kawanishi, Hyogo 666-0014 Japan; 3 Matsushima Clinic, 3-138 Isechou Nishi-ku, Yokohama, Kanagawa 220-0045 Japan; 4 Department of Gastroenterological Surgery, Osaka University Graduate School of Medicine, 2-2 Yamadaoka, Suita, Osaka 565-0871 Japan

**Keywords:** Colonoscopy simulator, Computed tomography colonography (CTC), Colonoscopy insertion method, Education and training

## Abstract

**Purpose:**

Laparoscopic surgery is now practiced widely because of its lower postoperative morbidity. As flexible endoscopy during laparoscopic surgery minimizes surgical trauma further, training in endoscopy will become more important for surgeons. Thus, we designed a physical simulator, the Noda–Kitada–Suzuki (NKS) model, which could provide the more realistic insertion of a colonoscope.

**Methods:**

We designed a colonoscopy simulator, based on information from computed tomography colonography scans of the anatomy and kinetic properties of the colon and rectum.

**Results:**

The transparent skeleton body of the NKS model provides instant visual feedback to the operator and the trainer. Our novel colonoscopy simulator replicates the realistic and reproducible insertion of a colonoscope from the rectum to cecum, providing authentic views of the Houston’s valves, the flexures, and mucosal folds. This was verified through an objective questionnaire, with 14 of 16 colonoscopists preferring the NKS model over the previous CM15 model for training purposes. Moreover, the Modified Colonoscopy Simulator Realism Questionnaire analysis confirmed that the NKS model was significantly more realistic than the CM15 for 7 (21.2%) of the 33 items when assessed by 12 colonoscopists.

**Conclusion:**

The NKS model provides a realistic training platform and may improve the quality of training in colonoscopy.

**Electronic supplementary material:**

The online version of this article (doi:10.1007/s00595-017-1517-7) contains supplementary material, which is available to authorized users.

## Introduction

Minimally invasive laparoscopic surgery has become widely used for the treatment of colorectal cancer and inflammatory bowel disease [1–3]. To further minimize the surgical trauma and postoperative complications [4], single incision laparoscopic surgery and transanal total mesorectal excision have been developed, and procedures combining flexible endoscopy and laparoscopic surgery, such as natural orifice transluminal endoscopic surgery (NOTES), are also receiving attention [5]. To utilise flexible endoscopy effectively during advanced and complicated laparoscopy, the surgeon must be skilled in performing conventional flexible endoscopy. However, the skills required to become proficient at performing colonoscopy can be difficult to teach and learn [6–10].

**Fig. 1:**
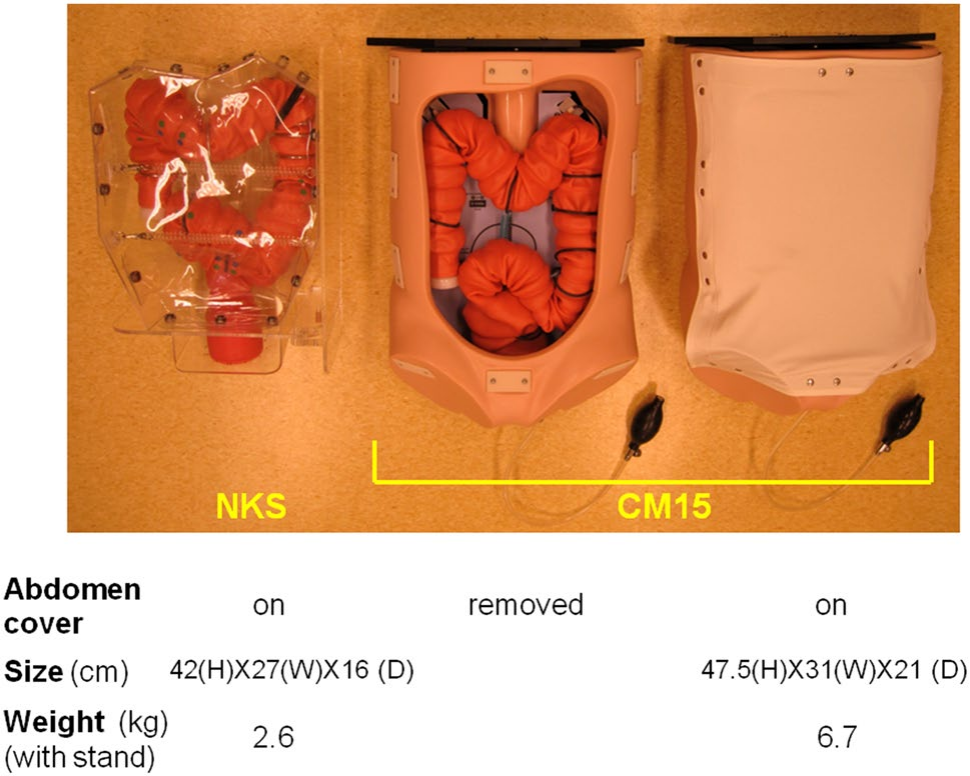
Overview of the Noda–Kitada–Suzuki (NKS) model and CM15 colonoscopy simulators. The NKS model is relatively light and has been designed to fit into a suitcase compact enough to be carried as hand luggage on aircrafts

The efficiency of colonoscopy training can be enhanced through practice with simulators, including simple physical models [9–13], physical models with interactive sensors [8], and computer-based virtual simulators [14–21]. These models are equally effective for acquiring basic colonoscopy skills [9–11, 13]. However, colonoscopy simulators are only moderately realistic compared with real colonoscopy [6, 7, 10, 11]. Thus, we designed a simulator that could offer more realistic insertion of the colonoscope. A novel physical model was developed to provide a training platform that was relatively affordable and accessible. The final product, the Noda–Kitada–Suzuki (NKS) model (Table 1), is an evolution model of an established simulator, CM15, the Colonoscope Training Model (manufactured by Kyoto Kagaku Co. Ltd., Kyoto, Japan; Fig. 1), which has already been validated as a credible simulator [9–12].

**Table 1:** Main features of the Noda–Kitada–Suzuki (NKS) colonoscopy simulator

1. The silicone rectal unit offers reliable and realistic endoscopic views of the Houston’s valves and the recto-sigmoid junction. It also supports realistic smooth insertion into the proximal colon
2. The sigmoid colon forms loops commonly encountered during real colonoscopy. The loops in the sigmoid colon are also resolved by maneuvers used during real colonoscopy
3. The three morphological features of the sigmoid colon can be pre-set with ease through simple steps
4. Similar to real life, the major movements in the transverse and sigmoid colon with postural change are prevented by the suspensory support and abdominal membrane
5. The transparent skeleton allows instant visual feedback to the operator and trainer
6. All the components are totally water-resistant for easy maintenance
7. The entire model is light and fits into a compact suitcase suitable to be carried as hand luggage on aircrafts

## Methods

### Development of the NKS model

#### CT colonography (CTC) images

We assessed the CTC scans of patients who underwent CTC imaging at Kawanish City Hospital for abdominal pain or altered bowel habits between June, 1 and December, 31, 2012, in accordance with the 1964 Declaration of Helsinki. Colonic distension for CTC was achieved with the automated continuous delivery of carbon dioxide. An 80-MDCT scanner (TSX-302A, Aquilion PRIME, Toshiba Med. Sys. Corp. Japan, Tochigi, Japan) was used for the CT images after standard bowel preparation. MDCT data were analyzed by a 3D image volume analyzer to obtain CTC images (VINCENT Ver3.3, FUJIFILM Med. Sys. Corp. Japan, Tokyo, Japan).

#### Silicone rectal unit

The simulator was developed at Kyoko Kagaku Co., Ltd. (Kyoto, Japan).

**Fig. 2:**
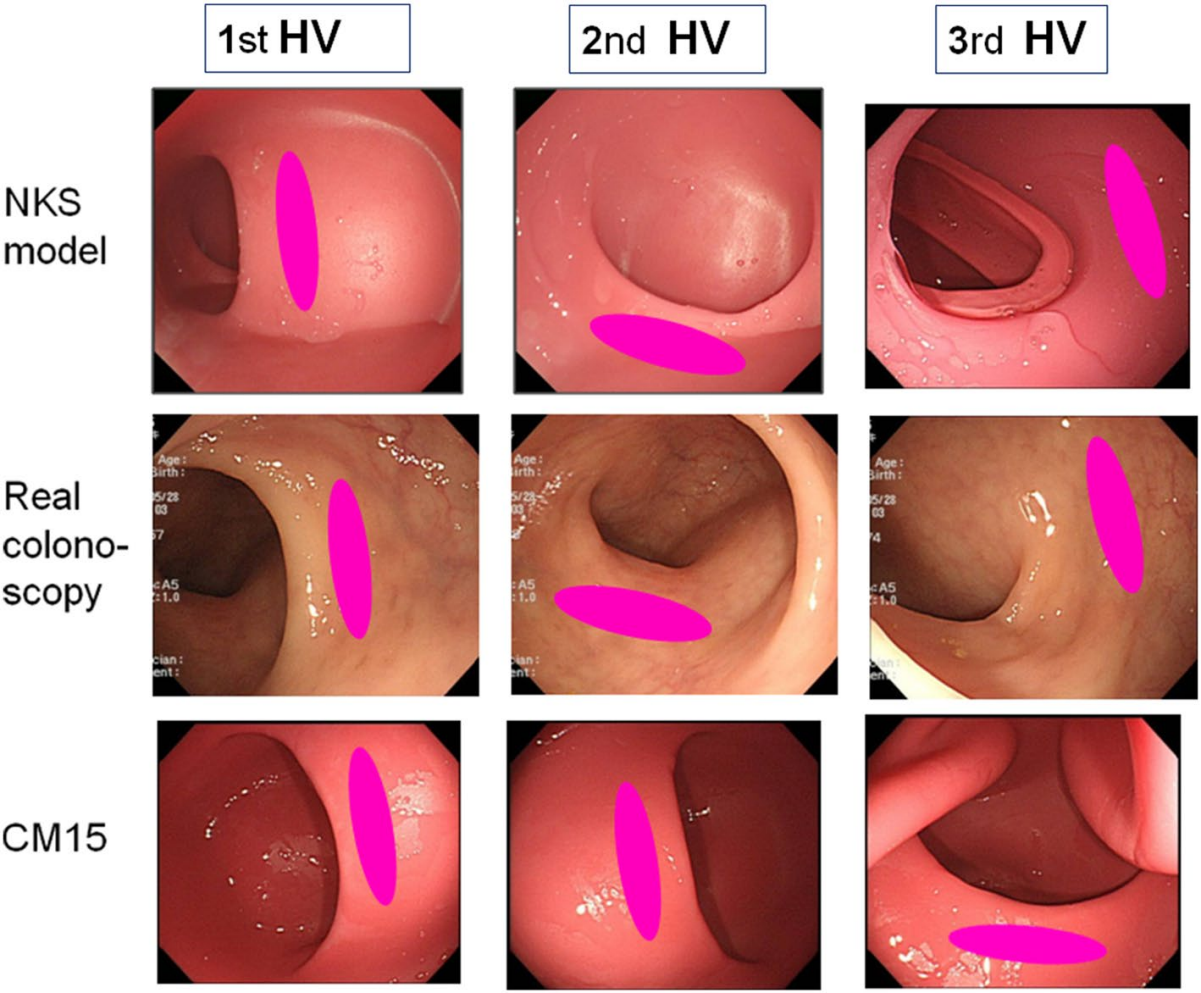
Novel silicone rectal unit designed with the aid of computed tomography colonography (CTC) images provides more realistic endoscopic views of the Houston’s valves than the CM15 model. All endoscopic views of the rectum were taken with these simulators and a patient in the left-lateral position. The photos in the upper panels are from the NKS colonoscopy simulator, the middle panels from the patient, and the lower panels from the CM15 model using identical insertion procedure. *1st HV* first Houston’s valve; *2nd HV* second Houston’s valve; *3rd HV* third Houston’s valve

The rectal unit of the NKS model is made of silicone and is an exact replica of the rectum of a patient, having taken dicom data from CTC images to create an acrylonitrile–butadiene–styrene resin cast with a 3D printer (Fortus 360Lmc-L, Stratasys, USA). The stiffness of the silicone in the current model reliably offers more realistic endoscopic views of the Houston’s valves and the recto-sigmoid junction than does the CM15 (Fig. 2).

#### Morphology of the sigmoid colon

**Fig. 3:**
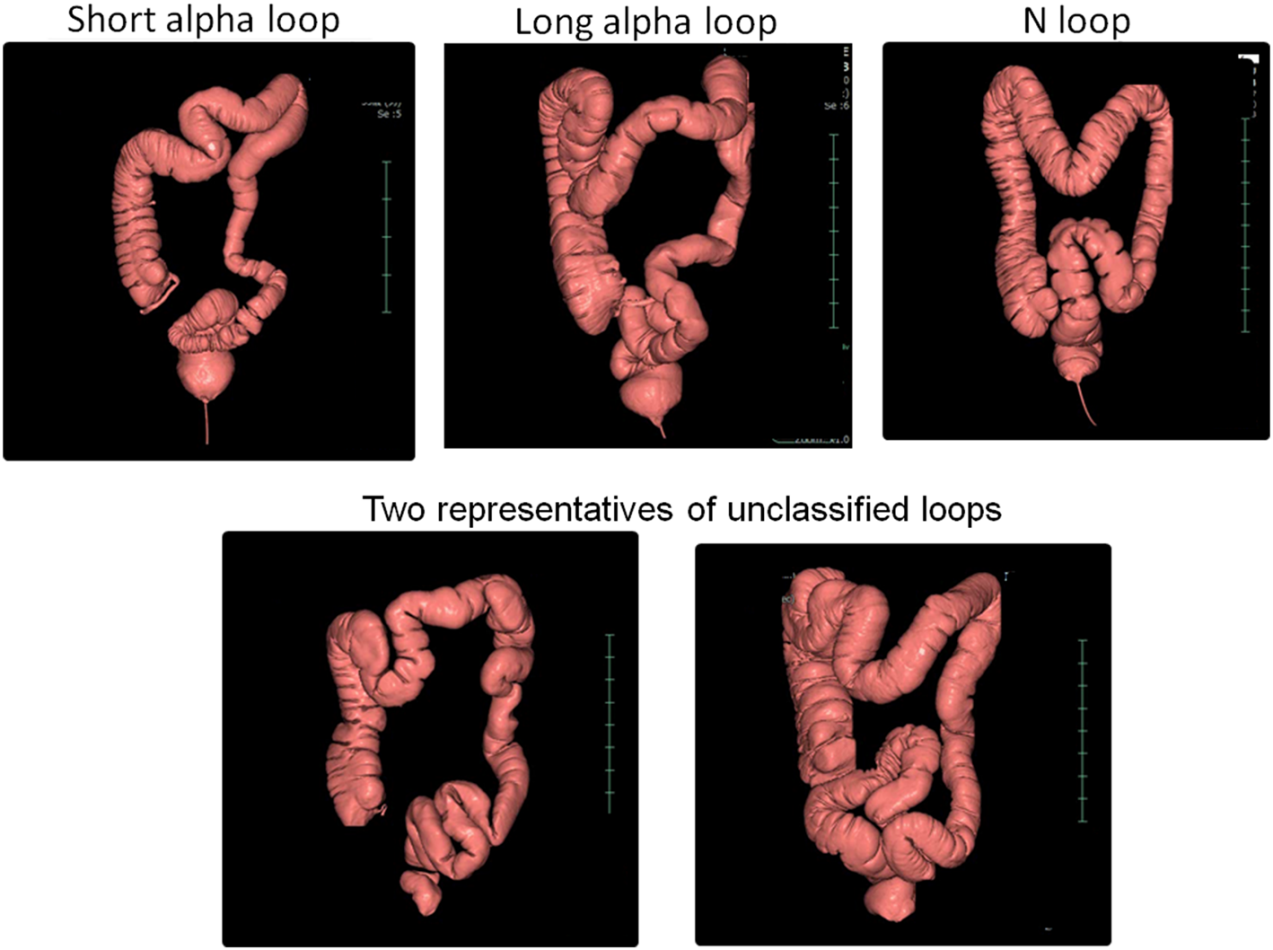
Three commonest morphological features of the sigmoid colon encountered on CTC. Short alpha loops, 15.2% (*n* = 16); long alpha loops, 24.8% (*n* = 26); N loops, 53.3% (*n* = 56); unclassified loops, 6.7% (*n* = 7)

The morphology of the sigmoid colon was assessed by analyzing the CTCs of 105 consecutive patients. Intriguingly, we found that the morphology of the sigmoid colon in the vast majority of the patients conformed to any of three morphological patterns: short alpha loops (15.2%), long alpha loops (24.8%), or N loops (53.3%) (Fig. 3). Based on these findings, the NKS model was designed, so that the sigmoid colon could be pre-set to take up any one of the three commonest morphologies. This was achieved by providing sufficient width and depth to the pelvis, as well as optimizing the suspensory and restrictive attachments to the sigmoid colon, which in turn allowed the sigmoid colon to move more naturally during colonoscopy.

The setting of the morphology could be interchanged easily by sliding the colon through its attachments, and then bending or twisting the colon into the desired position (Online Resource 1). As with real colonoscopy, the operators are unlikely to accomplish cecal intubation by merely using a continuous push technique, and must instead resolve loops that form and pass over the mucosal folds and flexures realistically (video recording, Online Resource 2).

#### Attachments of the colon

**Fig. 4:**
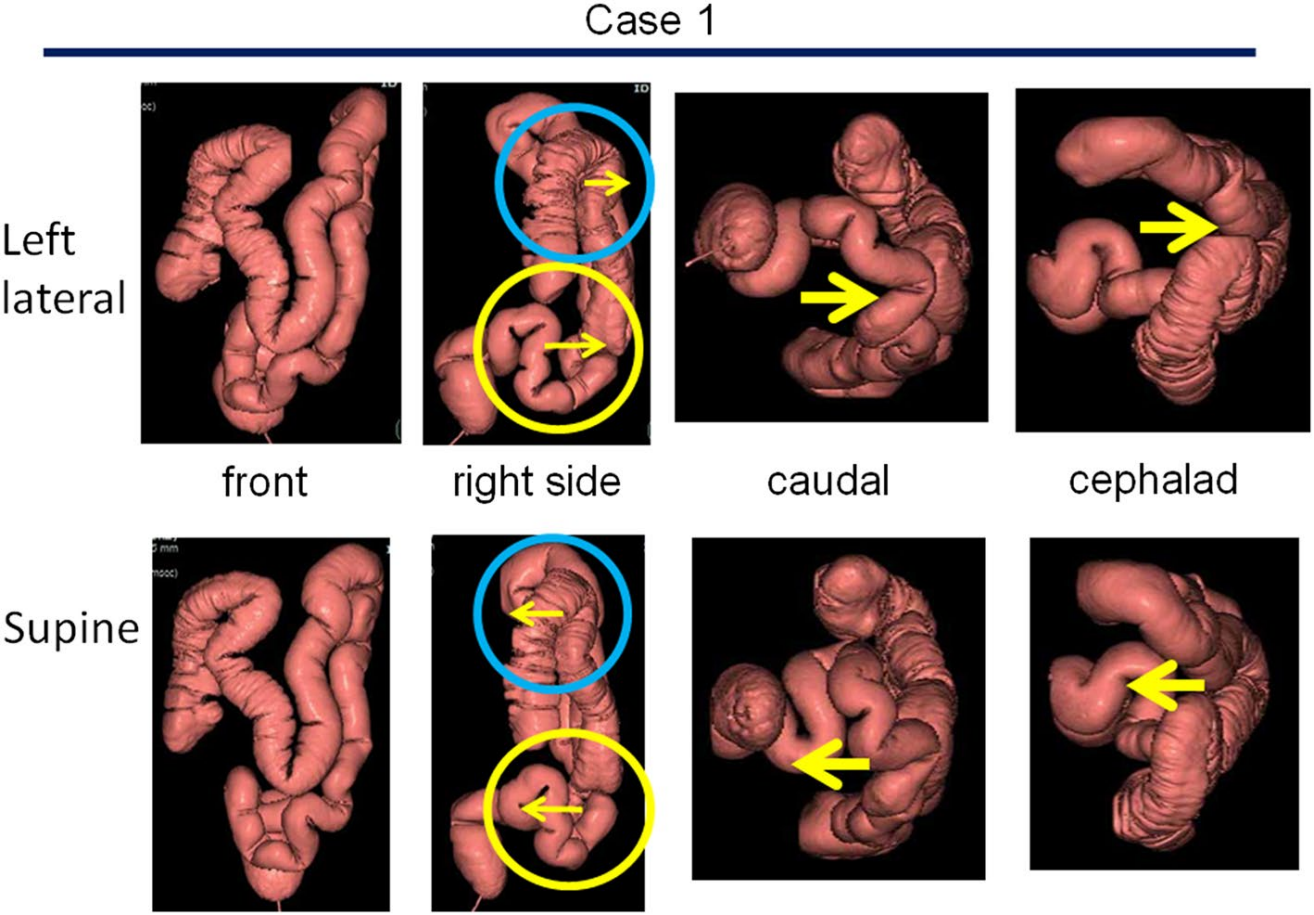
CTC images from a patient in the supine and left-lateral positions. The position of the colon changed minimally with the change in posture when assessed from the front in all 20 patients. On the contrary, as shown representatively in Case 1, the sigmoid-descending colon junction and hepatic flexure were loosened by the postural changes from the supine to the left-lateral position by forward-shift movements of the transverse and sigmoid colon

**Fig. 5:**
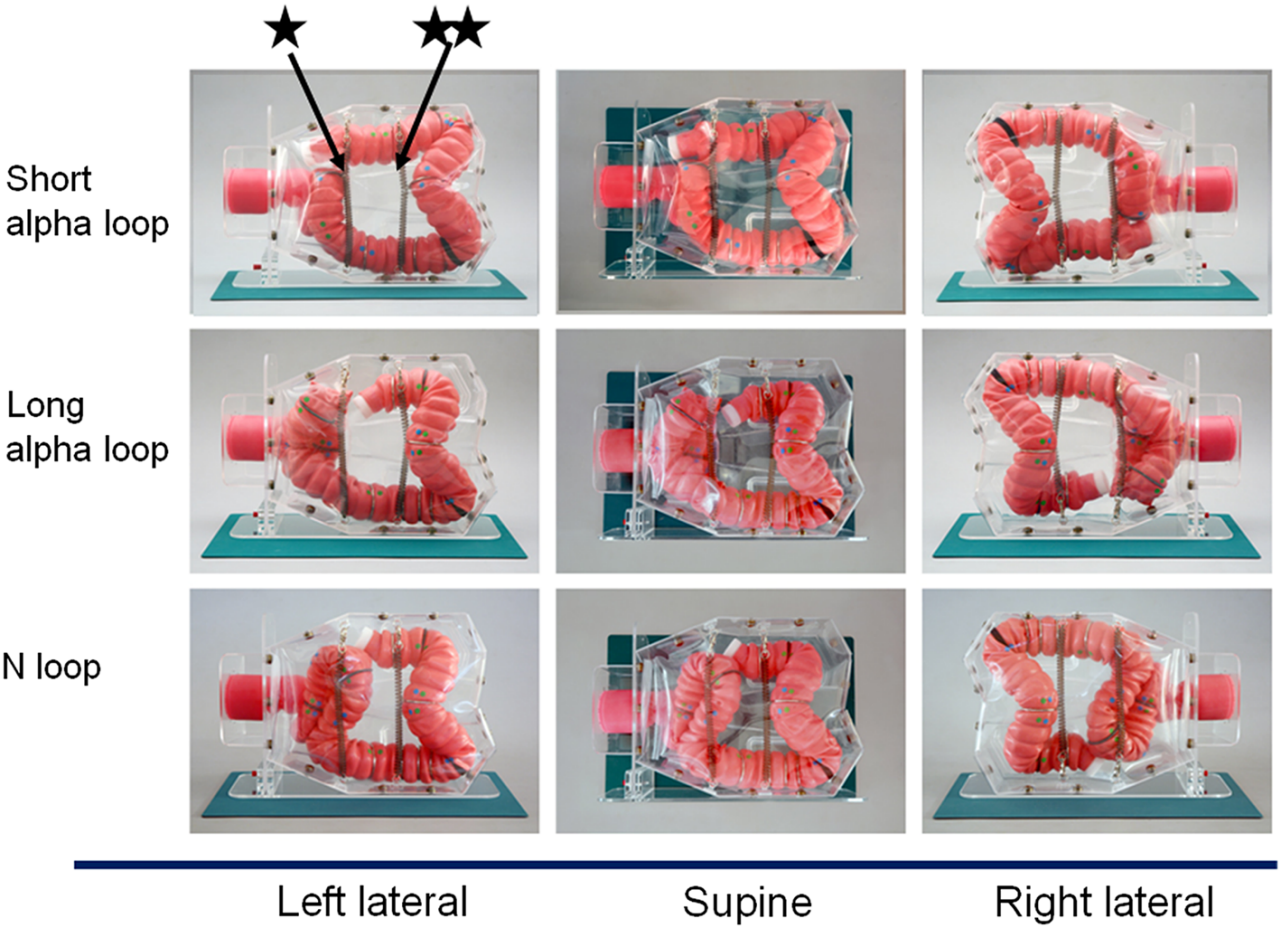
Introduction of new transverse and sigmoid colon suspensory attachments to reduce redundancy of the colon tube. The new suspensory attachments (*star*) and (*double star*), to the sigmoid and the transverse colon respectively, together with the transparent abdominal membrane, reduce excessive movement of the colon in any posture

CTCs from 20 of 105 patients who underwent imaging in the supine and left-lateral positions were analyzed to establish how the shape of the colon differed in different postures. There was relative loosening of the sigmoid-descending colon junction and hepatic flexure in the left lateral vs. the supine postures, but overall, the position of the colon did not change remarkably (Fig. 4; Online Resource 3). Suspensory supports for the transverse and sigmoid colon were, therefore, introduced, which together with the abdominal membrane, prevented major colonic movements with changes to the posture in the NKS model (Fig. 5).

#### Transparent body and model components

**Fig. 6:**
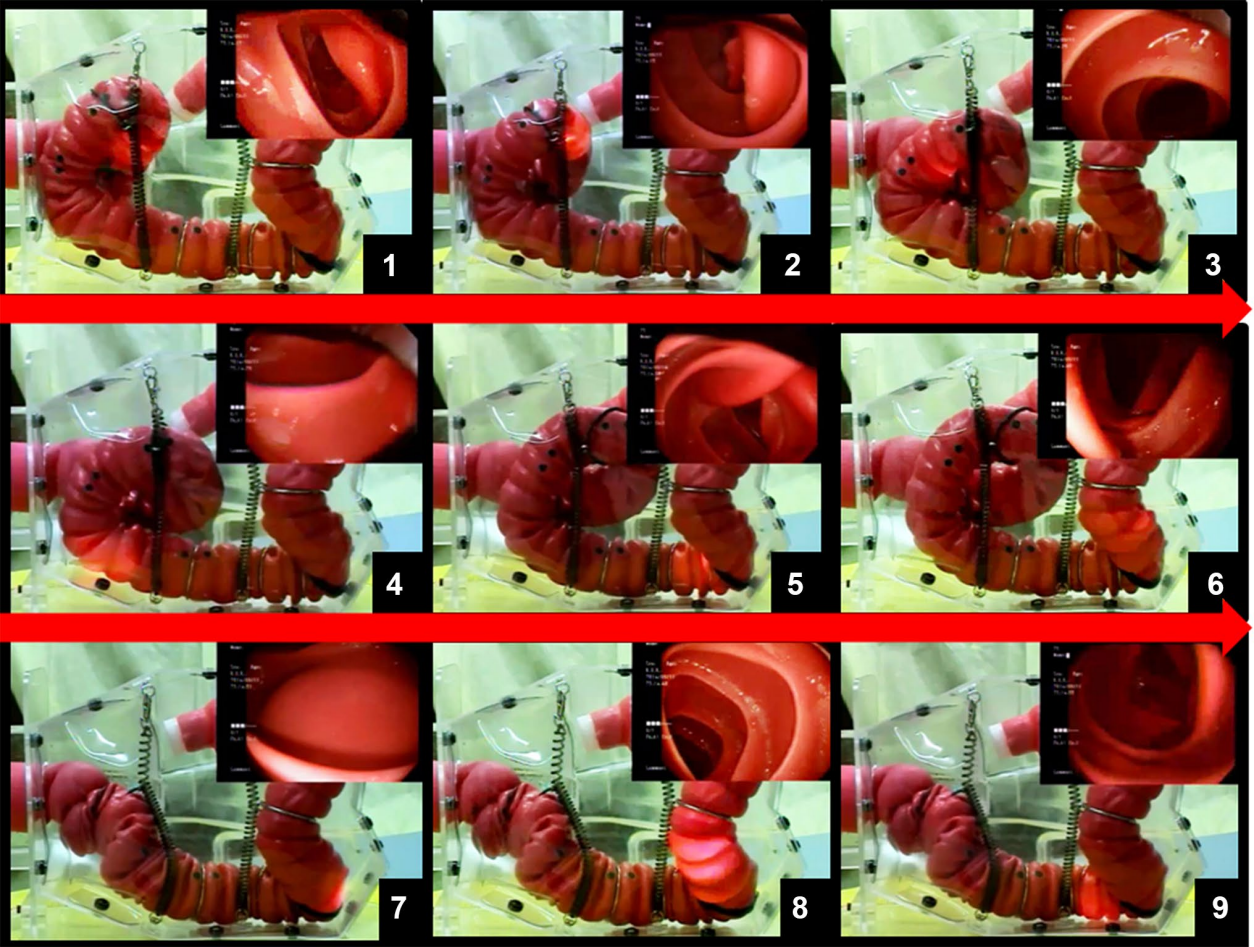
Time lapse photos of colonoscopy with the NKS colonoscopy simulator. This figure illustrates colonoscopy insertion in a sigmoid colon with a long alpha loop in the left-lateral position. The distal tip is maneuvered to pass over the splenic flexure into the left transverse colon by gentle pushing along with bending the scope tip upward (*5*–*6*). While maintaining the scope tip in the fully upward bending position, the long alpha loop is resolved by applying a clockwise torque with delicate retraction of the scope shaft (*6*–*8*). After the loop is resolved, the scope tip is released back to the neutral bending position. Then the distal tip of the colonoscope is intentionally retracted to the descending colon to ensure the scope shaft is freely mobile, using gentle pushing and retraction repeatedly (*9*). Thereafter, the colonoscope is inserted into the cecum (Online Resource 2)

Guided by the CTC images, the vertebral body of the NKS model was made to project more into the abdominal cavity, resulting in realistic endoscopic intubation through the recto-sigmoid junction and hepatic flexures. The skeleton body, abdominal membrane, and colon tube attachments are all transparent (Online Resource 4), which enables the operator to directly observe the intubation process and appreciate the forces delivered to the colon by the colonoscope (Fig. 6; Online Resource 2).

#### Maintenance and transportation

The NKS model was designed to be entirely water-resistant, so that it can be cleaned and maintained easily. The model is relatively light and fits into a suitcase that is compact enough to be carried as hand luggage on aircrafts (Fig. 1).

### Evaluation of the NKS model

The usefulness of the NKS model for training purposes was compared with that of the CM15 model, the most utilised physical simulator for colonoscopy training, by 16 colonoscopists from five district general hospitals, one university hospital, two private hospitals, and two endoscopic clinics, who completed a signed questionnaire. Fourteen of the colonoscopists were certified by the Japan Gastroenterological Endoscopy Society (JGES) and 2 were residents. The 16 colonoscopists included 5 very experienced colonoscopists with a record of 25,000–12,000 colonoscopies, 9 experienced colonoscopists (6000–1000 colonoscopies), and 2 less experienced colonoscopists (fewer than 300 colonoscopies). None of the participants declared a financial relationship with any company that manufactures or distributes colonoscopy training equipment. The recruitment and testing were conducted between March 19 and May 12, 2016. The colonoscopists evaluated the models with the sigmoid colon set in all three morphologies, including the short alpha loop, long alpha loop, and N loop.

#### Overall evaluations

Overall evaluations were based on the results of a questionnaire comprised of three simple questions; namely:

1. “Which would be more ideal for learning if you were an observer?”
2. “Which would be more helpful for learning to overcome the difficulties with the insertion of the colonoscope?
3. “If you have the opportunity, which one would you prefer to use?”

The colonoscopists were asked to choose their answers from the NKS model, the CM15, both, or neither.

#### Evaluation of colonoscopy simulator realism to real colonoscopy

Both simulators were evaluated for their realism using the Modified Colonoscopy Simulator Realism Questionnaire (M-CSRQ; Table 2), which consists of 33 items divided into seven prior subscales. The original CSRQ consists of 58 items divided into ten prior subscales, to compare specific aspects of the colonoscopy simulators [10]. Twenty-one items from the original CSRQ were not applicable and excluded, because they were designed for the evaluation of other forms of colonoscopy simulators, such as physical models with interactive sensors and computer-based virtual simulators equipped with/without a simulator colonoscope. Four of the items were excluded from the “*Visual*” subscale, since both the simulators were equipped with an identical colon tube, excluding rectum. All 33 items were rated from 1 (“extremely poor”) to 6 (“extremely well done”). Four colonoscopists from the original 16 were excluded from M-CSRQ analysis, because they did not answer large parts of the questionnaire.

**Table 2:** Modified Colonoscopy Simulator Realism Questionnaire (M-CSRQ) Analysis

Realism Sub-scale Items		*P* value	NKS		CM15	
			Mean Score	SD	Mean Score	SD
*3. Anatomical Structure*						
10. How realistic was the length of the colon?	n.s	0.58524	4.75	1.06	4.50	1.09
11. How realistic was the degree of angulation at the rectosigmoid junction?	*P* < 0.01	0.003329	5.42	0.51	4.00	1.21
12. How realistic was the degree of angulation at the sigmoid-descending colon junction?	n.s	0.06849	5.08	0.67	4.25	1.22
13. How realistic was the degree of angulation at the splenic flexure?	n.s	0.08384	5.25	0.75	4.58	1.00
14. How realistic was the degree of angulation at the hepatic flexure?	*P* < 0.05	0.03536	5.17	0.83	4.25	1.06
*4. Visual*						
21. How realistic was the appearance of the rectum?	*P* < 0.05	0.029249	5.17	0.94	3.92	1.38
22. How realistic was the appearance of the sigmoid colon?	n.s	0.18811	4.75	1.22	4.00	1.14
23. How realistic was the appearance of the descending colon?	n.s	0.32312	4.92	1.16	4.42	1.31
24. How realistic was the appearance of the transverse colon?	n.s	0.11827	4.92	1.24	4.25	1.06
25. How realistic was the appearance of the ascending colon?	n.s	0.24894	4.75	1.06	4.33	1.07
30. Overall, how realistic was the visual representation of the colon?	n.s	0.16612	4.92	0.90	4.33	1.07
*5. Visual Response*						
31. How realistic was the response of the visual image when you advanced the scope?	n.s	0.06909	5.08	0.90	4.33	0.89
32. How realistic was the response of the visual image to steering maneuvers?	*P* < 0.05	0.03657	5.17	1.03	4.33	0.78
47. How realistic was the response of mucosal folds to subtle steering maneuvers of the colonoscope?	n.s	0.07436	4.92	1.24	3.92	1.24
*6. Haptic Response*						
33. How realistic was the amount of forward insertion force required?	n.s	0.07672	4.50	1.38	3.67	1.30
34. How realistic was the amount of “torque” (clockwise or counter-clockwise rotational force) required?	*P* < 0.01	0.00685	5.17	0.72	4.00	1.21
35. How realistic was the feel of resistance to movement of the colonoscope shaft?	n.s	0.3269	4.08	1.31	3.58	1.24
36. How realistic was the feel of resistance to movement of the colonoscope steering controls?	n.s	0.11664	4.58	0.90	3.83	1.19
37. Overall, how realistic was the feel of resistance to movement of the colonoscope?	n.s	0.11096	4.50	1.09	3.75	1.29
*7. Insufflation and Deflation*						
38. How realistic was the visual representation of air insufflation?	n.s	0.17766	4.42	1.00	3.67	1.37
39. How realistic was the visual representation of air deflation?	n.s	0.25732	4.33	1.07	3.67	1.37
41. How realistic was the visual representation when suction was applied?	n.s	0.13747	4.42	1.00	3.67	1.23
45. How realistic was the response of mucosal folds to air insufflation?	n.s	0.29444	4.33	1.07	3.83	1.12
46. How realistic was the response of mucosal folds to suction?	n.s	0.32085	4.25	1.14	3.75	1.06
*8. Navigation Difficulty*						
43. How realistic was the difficulty of navigating the colonoscope around bends and angulations?	n.s	0.07409	5.00	0.95	3.92	1.51
44. How realistic was the difficulty of navigating the colonoscope around mucosal folds?	n.s	0.15238	4.83	0.94	4.08	1.31
*9. Looping*						
48. How realistic was the ease with which loops formed?	n.s	0.05985	4.75	0.97	3.92	1.16
49. How realistic was the extent of any looping that occurred?	n.s	0.12951	4.67	1.07	3.92	1.24
50. During looping, how realistic was the extent of any paradoxical scope motion?	n.s	0.15475	4.67	1.15	4.00	1.28
51. During looping, how realistic was the feel of resistance to movement of the colonoscope shaft?	n.s	0.05719	4.67	0.89	3.92	1.00
52. During looping, how realistic was the location within the colon of resistance and paradoxical motion?	n.s	0.12009	4.58	1.00	3.83	1.19
53. How realistic was the response of the simulator to loop reduction with typical techniques?	*P* < 0.05	0.0282	5.17	0.72	4.25	1.06
54. Overall, how realistic was the simulation of looping during insertion?	*P* < 0.05	0.0282	5.17	0.72	4.25	1.06

*SD* standard deviation

### Statistical analyses

Each subscale score for both simulators was statistically analyzed for mean and standard deviation. Finally, the difference in evaluation for both simulators to each item was statistically analyzed by a pairwise Mann–Whitney *U* test (StatMate V 5.01, ATMS, Tokyo, Japan). *P* < 0.05 was considered significant.

## Results

### Overall evaluations

According to the responses to the questions 1, 2, and 3, all the colonoscopists favored the NKS model, with the exception of one experienced doctor who answered “both”, and another experienced doctor who answered “neither” to the question 2.

### Evaluation of colonoscopy simulator realism to real colonoscopy

Both simulators were evaluated for realism using the Modified Colonoscopy Simulator Realism Questionnaire (M-CSRQ; Table 2). In 7 of the 33 items (21.2%), NKS was evaluated as significantly more realistic than the CM15 model, and as equivocal in the remaining 26 items. From the “*Anatomical Structure*” subscale, the degree of angulation at the “rectosigmoid junction” and “hepatic flexure” was evaluated as significantly more realistic in the NKS model (*P* = 0.003329 and *P* = 0.03536, respectively). From the “*Visual*” subscale, “the appearance of the rectum” was evaluated as significantly more realistic in the NKS model (*P* = 0.029249). From the “*Visual Response*” subscale, “the response of the visual image to steering maneuvers” was evaluated as significantly more realistic in the NKS model (*P* = 0.03657). From the “*Haptic Response*” subscale, “the amount of “torque” (clockwise or counter-clockwise rotational force) required” was evaluated as significantly more realistic in the NKS model (*P* = 0.00685). Finally, from the “*Looping*” subscale, “the response of the simulator to loop reduction with typical techniques” and “the simulation of looping during insertion” were evaluated as significantly more realistic in the NKS model (*P* = 0.0282, for both; Table 2).

## Discussion

There is a growing need for competent colonoscopists globally, and colonoscopy training is enhanced by the use of simulators [17–21]. It is envisaged that minimally invasive surgery will evolve to incorporate flexible endoscopy, which will require colorectal surgeons who are proficient at performing colonoscopy. Given that the existing simulators are considered only moderately realistic [6, 7, 10, 11], we were prompted to design a novel simulator to enhance colonoscopy training further. We took great care to make the current model as realistic as possible in terms of the loop formation at the sigmoid colon. The questionnaire survey confirmed that the NKS model was significantly more realistic than the CM15 model concerning “the response of the simulator to loop reduction with typical techniques” and “the simulation of looping during insertion”. These are important features of colonoscopy simulators, given that many, including ourselves, have emphasized the importance of resolving loop formations of the colonoscope prior to advancing the distal tip of the colonoscope much beyond the splenic flexure, to facilitate the rest of the intubation to the cecum being successful as well as comfortable for the patient [6, 7, 22–24]. With this novel rectal unit, an anatomically representative skeleton body and the arrangements of the suspensory and restrictive attachments of the colon, a simulator has been created to offer more realistic and reproducible intubation from the rectum to cecum as well as account for postural changes and application of abdominal pressure. Indeed, these accumulated refinements have resulted in a significantly more realistic anatomical angulation at the rectosigmoid junction as well as the hepatic flexure, and visual appearance of the rectum, as evidenced in the questionnaire evaluation. Although both simulators share the same colon tubes, excluding the rectal unit, these changes gave the NKS model significant advantages over the CM15, in terms of the response of the visual image to steering maneuvers and the amount of “torque” required for the haptic response (Table 2). The sigmoid colon can be set to any of the three commonest morphologies quickly and with ease, allowing the operator to spend more time on colonoscopy training (Online Resource 1).

The transparent body and supportive components of the simulator are unique among physical models, offering a simple yet effective means of feeding back real-time information to both the trainee and trainer to enhance the learning experience. The transparent abdominal cover of the NKS model also allows for visualization of the colon during practice in all positions, unlike the former CM15 model, which required removal of the opaque cover to obtain visual feedback, and was only possible in the supine position, since the other positions lead to extravasation of the colon during practice (Fig. 1; Online Resource 4). Footage of the colonoscopy being performed can also be stored for viewing later or transmitted live to an instructor in another location, or be used as part of an assessment (Online Resource 2).

The preference for the NKS model over the CM15 model for training purposes was confirmed by the results of an objective assessment by doctors with a wide range of experience, which may reflect the usefulness of the current simulator for learning the basic techniques and refining expert techniques. To at least partially overcome the risk of bias from conducting the present study using a small number of raters, we asked doctors from a number of institutions to evaluate these colonoscopy models. The current simulator also permits mild deflation of the rectum, but it does not allow full deflation given that it is made of stiff silicone. However, this did not seem to be an issue for the colonoscopists who evaluated the models and deemed both to be equivocal regarding “*Insufflation and Deflation*” during colonoscopy (Table 2). Although we cannot be certain that the morphology of the sigmoid colon we observed would be similar in a different population, it is consistent with the type of loops commonly encountered during colonoscopy [22–24].

Taken together, the NKS colonoscopy simulator may enhance a better understanding of the complex procedures of colonoscopy insertion, resolve the problems of the current colonoscopy training models, and improve the training for colonoscopy remarkably.

## Conclusion

The NKS model, which was developed based on our analysis of data from computed tomography colonography, provides a realistic training platform, and may improve the quality of training in colonoscopy significantly and cost-effectively. All these features are important for surgeons to acquire the necessary skills for performing colonoscopy proficiently.

## Electronic supplementary material

Below is the link to the electronic supplementary material.

Online Resource 1. The sigmoid colon can be set to the three most commonly encountered morphological features. The morphology of the sigmoid colon can be interchanged with ease, by sliding the colon into the position marked by colored labels, and then bending or twisting it into the desired conformation (TIF 920 KB).

Online Resource 2. Video recording of Fig. 6. The 1st HV, 2nd HV, and 3rd HV, the first, the second, and the third Houston’s valve, respectively; Rb, lower rectum (below the peritoneal reflection); Ra, upper rectum (above the peritoneal reflection); RS, recto-sigmoid; SD junction, sigmoid-descending colon junction (MPG 40767 KB).

Online Resource 3. Additional representations of CTC images from two patients in the supine and left-lateral positions. The position of the colon changed minimally with the change in posture when assessed from the front in all 20 cases. Two additional cases are shown representatively in this figure (supplementary figure for Fig. 4) (TIF 975 KB).

Online Resource 4. The NKS colonoscopy simulator. The transparent body and abdominal membrane provide a unique opportunity to understand and observe the forces caused by the colonoscope on the colon. The operator can also comprehend the proper application of abdominal pressure and postural change to augment intubation in difficult cases. Views from the (a) front, (b) back, (c) cephalad, (d) caudal, and (e) right (TIF 1629 KB).

## Abbreviations

NKS: Noda-Kitada-Suzuki
CM15 model: Colonoscope Training Model (manufactured by Kyoto Kagaku Co., Ltd. and distributed by Olympus Medical Systems Co., Ltd)
CTC: Computed tomography colonography
HV: Houston’s valve
SD junction: Sigmoid-descending colon junction
Ra: Upper rectum (above the peritoneal reflection)
Rb: Lower rectum (below the peritoneal reflection)
RS: Recto-sigmoid
