# Supplementary figures and images for: A novel physical colonoscopy simulator based on analysis of data from computed tomography colonography
Source: Surg Today. 2017 May 3;47(9):1153–62. doi: 10.1007/s00595-017-1517-7 (PMC5532419; doi:10.1007/s00595-017-1517-7)

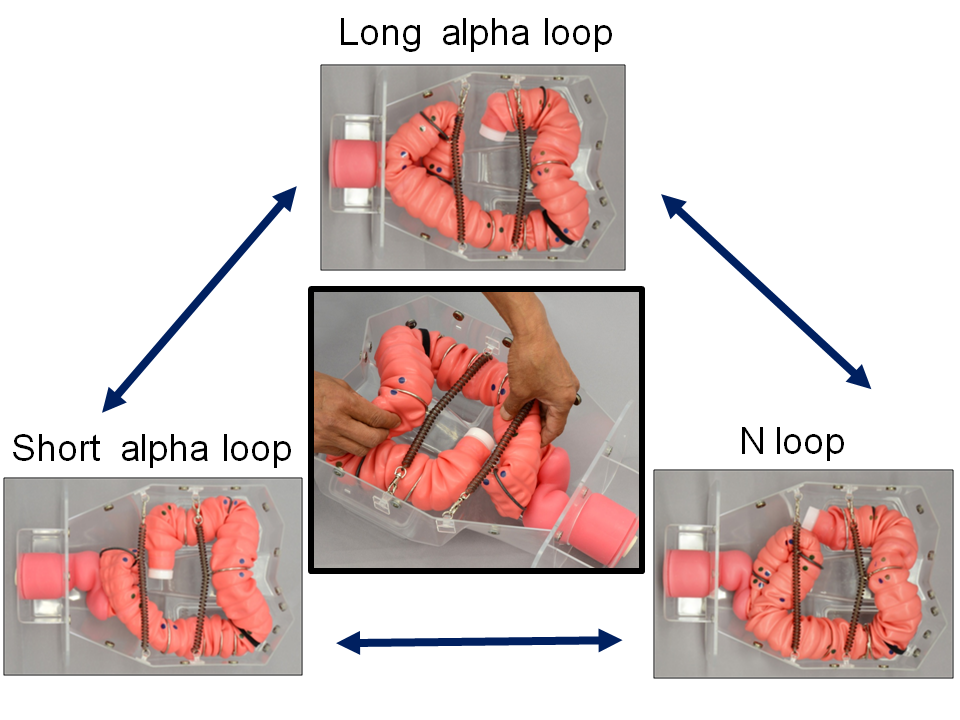

Supplement: Supplementary file 1 — Online Resource 1. The sigmoid colon can be set to the three most commonly encountered morphological features. The morphology of the sigmoid colon can be interchanged with ease, by sliding the colon into the position marked by colored labels, and then bending or twisting it into the desired conformation (TIF 920 KB). [file 595_2017_1517_MOESM1_ESM.tif]

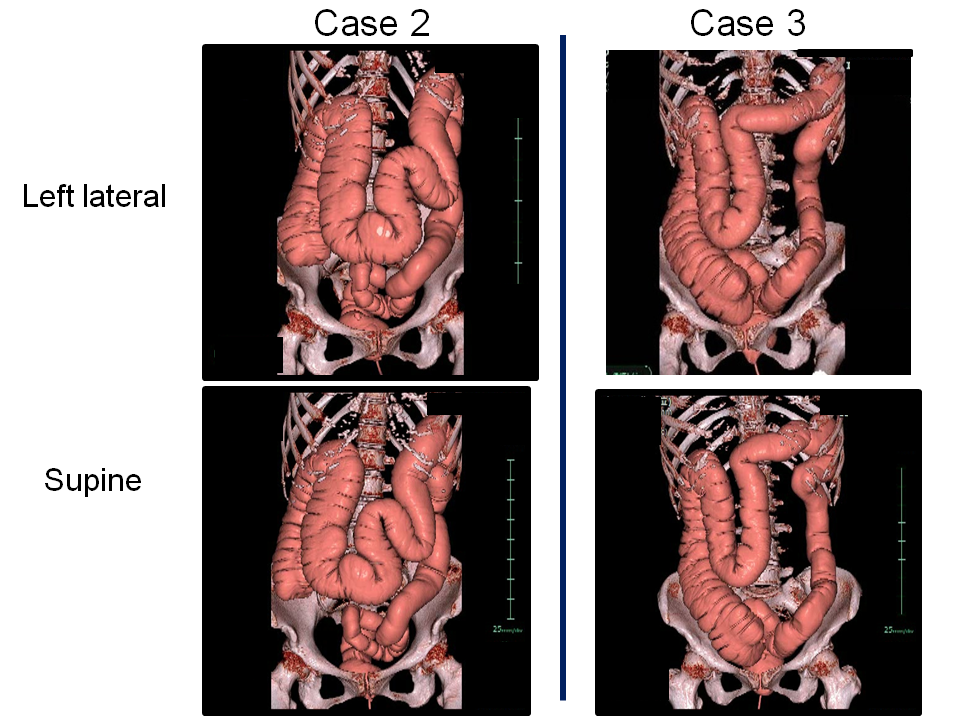

Supplement: Supplementary file 3 — Online Resource 3. Additional representations of CTC images from two patients in the supine and left-lateral positions. The position of the colon changed minimally with the change in posture when assessed from the front in all 20 cases. Two additional cases are shown representatively in this figure (supplementary figure for Fig. 4) (TIF 975 KB). [file 595_2017_1517_MOESM3_ESM.tif]

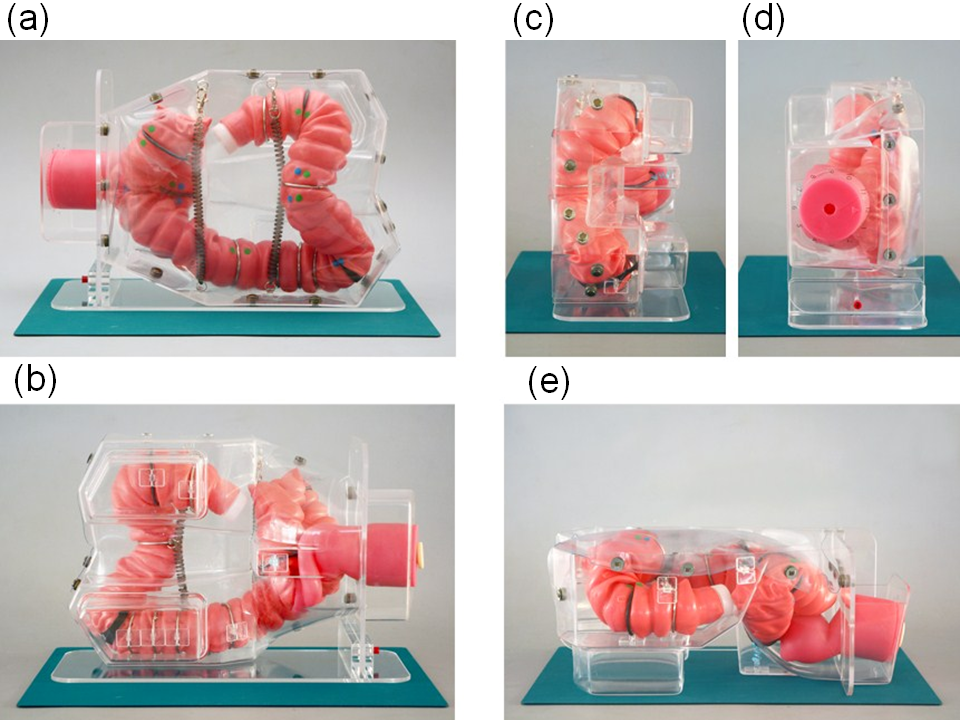

Supplement: Supplementary file 4 — Online Resource 4. The NKS colonoscopy simulator. The transparent body and abdominal membrane provide a unique opportunity to understand and observe the forces caused by the colonoscope on the colon. The operator can also comprehend the proper application of abdominal pressure and postural change to augment intubation in difficult cases. Views from the (a) front, (b) back, (c) cephalad, (d) caudal, and (e) right (TIF 1629 KB). [file 595_2017_1517_MOESM4_ESM.tif]
